# Assessing the Comprehensive Training Needs of Informal Caregivers of Cancer Patients: A Qualitative Study

**DOI:** 10.3390/curroncol30040291

**Published:** 2023-03-29

**Authors:** Janet Papadakos, Mohamed Ugas, Naa Kwarley Quartey, Christine (Tina) Papadakos, Meredith Elana Giuliani

**Affiliations:** 1 Cancer Health Literacy Research Centre, Cancer Education, Princess Margaret Cancer Centre, Toronto, ON M5T 3M6, Canada; 2 The Institute for Education Research (TIER), University Health Network, Toronto, ON M5T 1V4, Canada; 3 Institute of Health Policy, Management and Evaluation, University of Toronto, Toronto, ON M5T 3M6, Canada; 4 Department of Radiation Oncology, University of Toronto, Toronto, ON M5T 1P5, Canada; 5 Radiation Medicine Program, Princess Margaret Cancer Centre, Toronto, ON M5T 3M6, Canada

**Keywords:** cancer, caregivers, education, experience, relationships

## Abstract

Introduction: The increasing demand for cancer services is projected to overwhelm the cancer care system, leading to a potential shortfall in human resource capacity. Informal caregivers (unpaid family/friend caregivers of cancer patients) provide a significant amount of care to patients and the cancer care system could not cope without them. The aim of this study was to analyze the needs of informal caregivers (CGs) through interviews with cancer patients and CGs, and to assess the content and utility of a comprehensive caregiver training course. Methods: Cancer patients and CGs were recruited from an academic cancer centre to elicit their thoughts and perceptions of cancer CG education needs through a qualitative, phenomenological design using semi-structured interviews and a curriculum review activity. Results: Six patients and seven CGs were interviewed. Patients averaged 53.8 years of age and CGs averaged 53.1 years. Caregiver participants reported that they were unprepared for their caregiving role. Depending on the severity of the disease, CGs reported significant emotional strain. Most participants wanted more practical information, and all expressed the desire for greater social support for CGs. While there were differences in terms of desired modality (e.g., online, in-person), support for greater CG education was strong. Discussion: CGs experience a significant learning curve and receive little to no direct training or education to help them acquire the knowledge and skills they need to support a cancer patient. This is especially challenging for new CGs, for whom emotional and informational needs are particularly acute. Participants shared a great deal of endorsement for a comprehensive training course for new CGs. Given the multiple demands on their time, some participants suggested that consideration be made to establish synchronous classes. Participants held that having the course take place (online or in-person) at a specific time, on a specific date could help CGs prioritize their learning. Participants also endorsed the idea of “required” learning because even though CGs may recognize that a course could be beneficial, some may lack the motivation to participate unless it was “prescribed” to them by a healthcare provider.

## 1. Introduction

The increasing demand on cancer services (40% growth in Canada by 2030) [1,2] is projected to overwhelm the cancer care system where there will be a significant shortfall in human resource capacity. In Canada, one in three Canadians aged 15 and older are informal caregivers (unpaid family/friend caregivers of cancer patients) [3]. It is estimated that informal CGs (herein referred to as CGs) save the Canadian healthcare system CAD 25 billion annually in home care and other costs [4]. CGs provide a significant amount of care to cancer patients and the cancer care system could not cope without them; CG support will be even more critical in the future. CGs provide patients with medical (e.g., medication administration), instrumental (e.g., medical service coordination), tangible (e.g., daily living support), and emotional assistance [5,6]. Cancer is a complex condition where chronicity is certain, from dealing with the sequelae of treatments and, in some cases, end-of-life care [7]. Multiple studies demonstrate that the diverse supportive care needs of cancer patients and CGs are not being met. They face challenges physically (e.g., experiences with short-term and late side effects), psychologically (e.g., difficulty coping, fear, anxiety), practically (e.g., financial toxicity), socially (e.g., body image and relationship issues), and spiritually (e.g., questioning the meaning of life) [8,9,10,11,12].

Furthermore, patients and CGs are increasingly given self-care directives, with some requiring basic clinical and infection-control knowledge and skills [5,6]. When patients and CGs are not able to accomplish these self-care directives, clinical outcomes are negatively impacted. Support for cancer CGs is critical given this complexity and the non-linearity of the cancer journey, making caregiving responsibilities laborious and variable [13]. Improved recognition, integration, and support for CGs in mitigating the risk of becoming co-patients and the debilitating effects of CG burden are urgently needed [14]. CG burden is a growing issue that impacts the health of CGs and the cancer patient for whom they are providing care [15,16,17,18]. CGs are a vulnerable population, and commonly report feeling unprepared, distressed, burned out, depressed, and/or unable to continue caregiving for their loved one [16]. Additionally, CG engagement in self-preventive health behaviours is less likely, thereby increasing their risk of disease [15]. Poor CG well-being and perceived burden are significantly associated with patients’ risk for physical and functional impairments, for instance, swallowing and speech dysfunction in head and neck cancer patients [17] as well as poor quality of life [18]. Compounding this burden are the communication responsibilities between CGs and healthcare providers, as CGs are commonly integral to care coordination, treatment planning, decision-making, and pain management [19]. These considerations have a significant impact on the healthcare system.

The results of a review of training interventions for CGs of cancer patients show that the vast majority of programs for CGs are directed at psychological outcomes and do not holistically address the full spectrum of CG needs [20]. Although psychological well-being is critical to quality of life and effective caregiving, a wider range of CG needs are unmet [16]. In addition, most existing CG courses require in-person attendance at a specific time in a specific place. This type of in-person training limits access to those in rural areas (or geographically distant from the cancer centre), those with multiple care demands (children, etc.), and those with a lower socioeconomic status [21].

Despite the acknowledgement of significant unmet CG needs, a thorough understanding of what is needed to meet the education and training needs of CGs has not been explored in sufficient detail. The cancer system is approaching an impending crisis between the increasing prevalence of persons living with cancer, an oncology workforce shortage, and a resource-stretched health system [22,23]. In response to these pressures, oncology care is evolving by providing more complex treatment regimens in the outpatient setting (e.g., autologous and allogeneic transplants). These changes in oncology care place a greater responsibility on CGs [24]. Patients, and more so their CGs, are ill prepared for this transition in care delivery, particularly as CGs are expected to provide care requiring basic medical skills [6,22] over an extended period. Despite the high prevalence of cancer caregiving in Canada, and the significant need for prepared CGs, there are very few cancer CG skills training courses that are widely available [3] and the extant programs do not seem to adequately address all of the needs of CGs. The aim of this study was to analyze the needs of CGs by speaking with both CGs and cancer patients, and to assess the content and utility of a comprehensive CG training course.

## 2. Materials and Methods

The study employed a qualitative, phenomenological design, which aimed to describe and contextualize the experiences of groups and individuals so as to provide comprehensive information to inform best practices [25]. We invited participants to describe their experience of cancer and the caregiving process, and then utilized this feedback to inform and orient our understanding of caregiving as a phenomenon and the needs of cancer caregivers.

The researchers recruited cancer patients and CGs from a large academic cancer centre in Toronto, Canada. Potential participants were approached by clinicians acting as recruiters, and invited to take part in a single, 60-min. interview to elicit their thoughts and perceptions of cancer CG education needs through a semi-structured interview, and to share their thoughts on a proposed curriculum for a CG training course through an activity. The interview process was informed by the concept of reflexivity, whereby the interviewer was actively involved in the gathering of information by considering their own assumptions and subjectivity, and how this informed their analysis [26]. The interviews were conducted by co-author M. Ugas, a male researcher employed by the cancer centre and who possessed no personal experience with caregiving or cancer. Participants were not given any further information about the interviewer and were unfamiliar with him prior to their recruitment. The curriculum review activity involved asking participants to review a curriculum outline that the study team developed based on cancer CG needs reported in the literature. The curriculum was divided into three domains: managing the medical aspects of illness; managing changing roles in relationships to accommodate illness; managing the psychological consequences of illness through the use of problem-solving coping strategies (see Supplementary File S1). Once the participants reviewed the curriculum outline, they were asked to share their thoughts on it, in detail.

Patients and CGs were introduced to the study by a member of their healthcare team either in-person or virtually. Recruitment was conducted among patients and caregivers in various clinics, including the head and neck clinic, breast cancer clinic, and the allogeneic stem cell transplant clinic. For participants, the inclusion criteria required they be at least 18 years of age, and able to read, write, and speak English. Patients had to have been diagnosed with cancer and received at least one cycle of chemotherapy or targeted therapies, and/or radiotherapy treatment, and/or cancer surgery. Caregivers were simply defined as someone caring for someone diagnosed with cancer, without restrictions on time commitment. Participants were excluded if the patient was treated with curative surgery alone.

Informed, written consent was obtained from each study participant. Prior to the interview, participants were asked to complete a short questionnaire that consisted of 11 questions concerning demographics such as age, gender, race, income, education, marital status, living arrangements, and the type of cancer the patient was diagnosed with. The questionnaire also included a validated 16-item health literacy assessment tool, the European health literacy survey questionnaire, and one 12-item computer literacy scale, the computer proficiency questionnaire [27,28]. These metrics were used to assess the capability of participants to understand, appraise, and research health information.

The semi-structured interview guides consisted of 26 questions that began with a series of warm-up questions that broadly asked participants about their experiences with cancer and with caregiving. Once the warm-up was complete, the interviewer asked a series of questions related to cancer health literacy at the point of diagnosis to gauge participant knowledge of cancer. Following this part of the interview, participants were asked to talk about how they learned about caregiving-related knowledge and skills (e.g., the disease, treatments, and symptom management). The interviewer probed to ask what the interviewee considered to be the most relevant information for themselves and what they considered to be the most relevant information for future caregivers. Participants were also asked how they would suggest future caregivers acquire this information. The next questions asked about the emotional and physical impact of caregiving and were followed up with questions about what strategies they might recommend to future caregivers to help them cope. Finally, participants were asked for their thoughts on a curriculum outline for a caregiver course designed to help them prepare for the role. If the response was affirmative, follow-up questions were asked regarding when the course should be offered, what content should be included, and how it should be delivered (see Supplementary File S1).

A maximal variation purposive sampling strategy [29] was used to generate a heterogeneous group of participants to capture a broad range of perspectives on characteristics of the following: age; cancer type; education level; treatment type (chemotherapy and/or radiotherapy, multimodality, targeted); living situation: alone, rural, or urban location; race; and varying levels of computer and health literacy.

Audio recordings of the interviews were transcribed verbatim. Participant demographics were summarized using descriptive statistics. Themes were coded inductively using the software program Nvivo by the interviewer based on the transcripts and field notes [30]. Feedback shared about the curriculum outline was analyzed in the same manner as the interview transcripts. Participants did not provide feedback on the findings, nor were they given access to transcripts. Data saturation was determined based on the degree to which the coding process was returning the same or similar information and themes. At this point, no further recruitment was conducted.

Internal ethics approval was obtained. Upon completion of the study, participants were thanked with a CAD 5 gift card as a token of appreciation. The results of this study were used to inform the content of a newly launched caregiver education platform that is currently undergoing phase 2 evaluation.

## 3. Results

Interviews were conducted from February 2021 until December 2021. A total of 21 participants were initially recruited. Eight were lost to follow-up for various reasons such as lack of time to participate and deteriorating prognosis. One patient passed away. This left a total of 13 participants who completed the interviews. Of these, seven were CGs and six were patients.

### 3.1. Participant Characteristics

Patients averaged 53.8 years of age, with participants ranging from 43 to 70 years. CGs averaged 53.1 years, with a range of 29–71. Most participants were male, white, and reported their highest level of schooling as some college or university education. The majority lived with a partner or children (*N* = 9) and were working or receiving disability payments (*N* = 10). Nearly half (*N* = 6) of participants reported a household income of greater than $100,000 in the past year. The types of cancers represented were eleven head and neck cancers and two breast cancers. The caregivers were either spouses, children, or siblings. CGs outscored patients on the health literacy measure, averaging 52.7/64 compared to 45.8/64, as well as on the computer literacy metric, scoring an average of 46.7/48 compared to 42.7/48 (Table 1). While we did not collect information from participants on the stage of the cancer care trajectory they were in, through the course of the interviews our participants shared a range of experiences from diagnosis through to end-of-life and survivorship.

### 3.2. Thematic Analysis

Having no experience with caregiving in the context of cancer, but being part of the team developing and building the caregiver support program, the interviewer and coder needed to remain cognizant of their hopes for endorsement of the curriculum so as to minimize any undue influence on the direction of the interview. Thus, the interviewer endeavored to stay attuned to what participants shared with respect to the support or challenges they experienced that were not related to education/training, as well as remaining open to receiving any critical feedback on the draft curriculum.

Three overarching themes were identified in the thematic analysis: 1. The lack of preparedness for the role of being a cancer caregiver; 2. The differing approaches to information and research among CGs; 3. The desire for greater social support among CGs, especially the need to speak with those with similar experiences.

#### 3.2.1. Caregiver Preparedness and Duties

Participants reported that there were a myriad of duties that caregivers would fulfill including assisting with medication, bandages and dressing, bathing, and more. The extent of these duties varied greatly and were usually dependent on the degree of autonomy the patient had. CGs for patients with high levels of autonomy reported duties limited to driving the patient to appointments, while CGs who cared for patients with more extensive needs completed tasks related to the patient’s care as well as other household tasks, particularly those that the patient had once assumed but was no longer in a position to do. For example, one patient participant shared examples of their caregiver’s responsibilities:

*“(My caregiver looked after) Making sure I had the proper appointments, the medication ready, hooking me up to the G-tube, ensuring my feeding was on track. Driving me to my appointments.”*

CGs reported that some of these duties were new to them, and most felt unprepared for the time commitment that comes with caring for a cancer patient. Some participants reported that, over time, their practical skills were sharpened as they utilized them, such as assisting with symptom management.

Related to symptom management, participants acknowledged that hospital resources did not always adequately address all the contingencies that could arise and the range of issues the patients could experience.

With regard to how to prepare to be a CG, one CG participant shared,

*“There’s no way of knowing this (i.e., the knowledge required to be a caregiver). There’s no way of reading about it that can prepare you because I believe every patient is unique and different. And so, their needs are different at any given moment in the day.”*

While participants argued that there was no substitute for experience and that experience helped to clarify information, they also regretted not knowing things sooner. As one caregiver explained:

*“I would have liked to have known a bit more before. I was sort of learning along with him [i.e., the patient] and it probably would have been helpful if I had known some things ahead of time to reassure him or help him go through the information because I’d already heard it, or knew of it, or talked about it.”*

On all matters related to CG preparedness and duties, participants expressed the need for good communication and cooperation between patients and their CGs. They stressed the importance of remaining open to each other’s concerns as well as being patient. They were especially concerned that others did not neglect the reality of the burden placed on CGs so as not to wear them out.

*“Patients (are) going through a lot … but not everyone (patients) is that good at active listening these days,”as one CG put it.*

CGs acknowledged that their primary concern was for the patient but wanted emotional support for themselves as well. In the words of one CG:

*“Caregivers really put themselves at the end of the road. They put themselves last and like the, you know…you don’t even know that you need help because you’re so entrenched and involved. (Because you want) to do right by the person you love, by your friends, your family, your neighbor, you know, whoever you’re caregiving and you want to be optimistic. And then, you know, you’re the last one in your own mind, because you think that’s the right thing to do, or you just get so swept up that you forget about you.”*

#### 3.2.2. Information Seeking and the Impact of Emotion on Learning

Most participants knew very little about cancer as a disease prior to the patient’s diagnosis. Those who did usually had a close relative who had cancer and their knowledge was related to their proximity to that patient, particularly those who acted previously as caregivers. One CG shared,

*“Well, you have to understand that you always think it’s a death sentence when you hear that word (cancer), you don’t think that no matter what people say to you, it is a horrible word. So, you have to figure when you first find out, you just think that this is over because you don’t hear a lot of success. You don’t hear the, the success stories, you only unfortunately hear the worst. So that’s where I struggled when I first found out.”*

As a result of this, some participants reported being overwhelmed by the information they received at the start of diagnosis. Views toward cancer information were either regarded as a tool or an impediment, with some preferring to acquire as much information as possible and others relying strictly on the actionable information necessary for decision-making. Participants also pointed to information-seeking on the part of CGs as one more way to alleviate the burden patients face.

*“So, my doctor gave me a package and the nurse said, I’d like you to read everything and look everything up. And I looked one thing up and then I closed the package and I went, ‘nope’, because I found I got high, high anxiety,” one patient explained.*

With patients having to process the emotional and physical ramifications of a cancer diagnosis, information-gathering was viewed as a role caregivers could easily assume to minimize the pressures patients face, while providing another trusted lens from which to view information and provide counsel.

It was especially true that patients required assistance with digesting information during the early stages of the cancer journey, when newly diagnosed patients and their caregivers receive the most information.

*“So, it really is a learning curve for caregivers. There’s no way to understand what this means. If no one sits down with you, throwing someone a welcome package with a bunch of information that may or not transpire in the life of the patient, it means nothing… it’s the caregiver that needs their hand held through the process.”*

Participants readily acknowledged that there was a cornucopia of information available from hospital resources and online and regarded it as a pragmatic decision to limit their information consumption to avoid becoming overwhelmed. Reading all available material made the retention of information difficult due to the sheer volume of it.

Patients also found that curtailing the amount of research they conducted on their own allowed them to avoid having to evaluate the quality and veracity of informal sources of information. This is particularly noteworthy due to the prevalence of misinformation on the Internet.

*“So, to me to be clear, I only researched questions that we should be asking when the doctors sent information home… I researched, you know, keywords and documents that they provided. So that way we understood what was being provided to us. But I know from my sister’s experience that, you know, doctors discourage people from going on the Internet and doing a whole bunch of their own research and [therefore I used] the doctor as really the conduit to gain information. So, my research was more about getting clarity about what the doctors provided. And I did find a couple of blog sites for men who had experienced the same type of cancer to get an understanding of what they experienced and questions that they asked and stuff like that.”*

There was a concern, however, among some CGs that they needed to know more information so that they did not feel like they were relegating decision-making to their healthcare team.

*“Some people just sort of leave everything to the doctors and don’t wanna know what’s going on. But I really wanted to know so I hopped on to Google and, you know, try to learn copious amounts information and like really quickly. And then I just talked to the doctors once I arrive(d).”*

#### 3.2.3. CG Need for Peer Social Supports

Participants all pointed to the emotional toll that a cancer diagnosis would exact on a patient and their loved ones. Multiple participants noted that during active treatment, when emotional strain was particularly acute, the psychological effect inflicted on a caregiver generally did not receive the attention they felt it deserved. CGs also, however, felt that their emotional well-being should be relegated to that of the patient and reported the desire to project optimism in the face of disease. Ultimately, this emotional labour contributed to feelings of isolation and loneliness. All participants, hence, reported the desire to connect with other caregivers, both for practical advice, and to fulfill a desire for understanding and community. Participants felt that CGs were not adequately prepared to have difficult conversations with loved ones that arose due to a cancer diagnosis, particularly those related to the possibility of death. This was in spite of feelings of fatalism among patients and their caregivers. One participant explained the initial views of patients such as herself:

*“The fact that they’ve been diagnosed with cancer is itself such a shock. And it’s hard to wrap their minds around the possibility that they could survive it.”*

CGs reported different methods for coping emotionally with the strain of caregiving. For most, this meant that engaging in activities, such as physical exercise, allowed them to not dwell on the looming possibility that the patient’s outlook may not be good. CGs reported that this blocking strategy was particularly necessary if the patient’s situation was deteriorating, and death was a distinct possibility.

*“It’s never ending and, it’s like bad news after bad news and you say, ‘okay, when is it going to stop?’ And if it’s going to stop or are we going to find a treatment to heal or at least to help you… you have pretty dark images in your head and even if you don’t want to get there, you go there.”*

Participants did note, however, that the extent of the burden placed on caregivers was related to the degree of autonomy maintained by the patient. This usually meant that patients with good prognoses did not require a great deal of time commitment from CGs.

### 3.3. Curriculum Feedback

With respect to a course for CGs, all participants endorsed the need for a comprehensive training course for CGs, but their views differed on its feasibility. The responsibilities and constraints on CG time posed barriers to in-person instruction. CGs had schedules that were routinely relegated to the needs of patients, which could change on short notice. Most participants recognized the value of virtual modalities for expanding access to the course, while also being keen to highlight the social/networking benefits of in-person learning. Virtual courses that are also synchronous may provide an ideal blend of the benefits of in-person instruction while maintaining the ease of accessibility necessary to ensure participation.

Participants were enthused by the draft CG curriculum they viewed. However, they expressed concerns over the vast quantity of the included information, pointing out that some of the information was relevant to them while some did not reflect their needs. This usually led participants to suggest some type of tailored module for CGs.

While there was a desire for flexibility, participants also pointed to the need to be held accountable, reasoning that attendance would benefit from in-person instruction by instilling greater commitment to the class. Some participants even called for mandatory attendance and some sort of evaluation that would help CGs to prioritize their learning. Participants, however, were generally unsure of how to encourage enrollment since they were already enthusiastic, with the benefit of hindsight:

*“I really don’t know how to motivate them. Like if it was something like that for myself, I would definitely (take it). I didn’t need to be motivated. I would definitely participate. I would definitely take the course. I mean, it depends on the individual, how involved they want to be.”*

Crucially, participants viewed in-person learning as a means to make connections with other CGs, which was something all participants highly supported as both a source of knowledge as well as emotional support.

## 4. Discussion

With the prevalence of chronic disease expected to rise, the support of informal caregivers will only become more important to the healthcare system [31]. Our findings confirm that cancer CGs experience a host of unmet education and support needs. The fractured nature of existing resources and programs for caregivers produces varying levels of satisfaction, usually relating to the severity, prognosis, and outcomes the patients face. CGs therefore are supportive of efforts to provide them with the tools they need and have endorsed the concept of a comprehensive training course.

Our sampling method was designed to generate a heterogeneous group of participants reflective of the diverse local population. Our participant population, however, was ultimately well-educated and of a generally high socioeconomic status. Health and computer literacy scores were similarly high. This may reflect selection bias, where the efforts of healthcare providers, who conducted recruitment, may have been skewed toward individuals whom they thought would be more likely to participate and provide thoughtful interviewees rather than focusing on demographic information. Participants with lower socioeconomic status and health literacy may not share these perspectives and prefer, for example, asynchronous learning, as it might provide greater flexibility to individuals who are unable to take time away from work.

Previous studies point to the extent of strain placed on caregivers. CGs continue to face acute challenges dealing with the burden of their new role, with little time to transition into it. They experience mental and physical strain, resulting in a deteriorating quality of life [31]. CGs of cancer patients report unmet supportive needs during both the active treatment phase as well as the post-treatment phase [32]. While the burden of caregiving is present throughout the disease trajectory, there exists a great deal of variation in how burdens manifest, pointing to the need for tailored solutions [33]. Research also shows that caregivers are receptive to online classes and the majority support asynchronous learning [34].

Our findings point to the central role of caregivers as conduits for information. Cancer care requires coordinating efforts from a multidisciplinary team of oncologists and other professionals, making the role CGs play in taking notes and synthesizing information crucial [35]. The roles of caregivers closely resemble the role of a knowledge broker by obtaining information, appraising it, and facilitating its exchange across a social network [36]. Caregivers have to work with different stakeholders and establish trust among them, which is another key component of being a knowledge broker [36]. In particular, they must have the trust of the decision-makers who rely on them to sift through vast quantities of information and support in identifying the implications of decisions, making their knowledge an important factor in patient satisfaction [37].

There was a great deal of variation among our participants regarding what the information needs of caregivers were. While the categorization of health information-seeking behaviour as “seekers” and “avoiders” has been criticized as overly simplistic, as individuals may transition between the two types depending on their needs [38], our participants broadly fit these groups with most either consuming as much information as they could or as little as possible. Caregivers usually adopted an information-seeking strategy that broadly complimented that of the patient, with a “seeker” researching more to alleviate the knowledge burden from an “avoider” patient. Concerns over the decision-making process did not materialize in the course of the interviews, as most participants felt sufficiently engaged by healthcare providers. Most of the cases in our study consisted of patients who were in remission; however, and according to Monteiro et al. it is when a patient’s condition deteriorates that the CGs exhibit greater deference to physicians [39]. Older populations such as ours, according to the literature, are also less likely to report dissatisfaction with healthcare providers [40].

Studies also tend to group CGs into different archetypes (lone caregivers, carrier caregivers, partner caregivers, and manager caregivers) and note the differences in information-seeking behaviour and health literacy among them [41]. Our more affluent and educated participants may have resulted in more manager CGs, for instance, characterized by their desire for medical information resulting in greater support for a comprehensive training course. Furthermore, if we had recruited more partner CGs, defined by their use of social support, we may not have generated the enthusiasm we did for CG psychosocial supports and training for how to develop coping strategies.

Those participants who reported the least unmet needs primarily cared for patients with significant autonomy, limiting their own burden, which is a finding that others have also reported [42]. The greatest unmet need was in the psychosocial domain, which supports the need for psychosocial education for caregivers. All participants reported requiring some sort of psychosocial support, with the exception of one highly spiritual CG, a characteristic associated with less distress among cancer CGs [43]. Our participants were also fairly health-literate, with CGs scoring slightly higher than patients. Patients with low health literacy are more likely to receive support from a CG [44]. The effect of CG health literacy on patient health literacy and health outcomes is unclear [45]. Health literacy among CGs is an area that has been cited as requiring more study [45]. While our study population had high educational attainment and good self-reported health literacy scores, they revealed in the process of being interviewed that research and knowledge synthesis on a topic as vast as cancer posed a considerable challenge for them.

Our sample consisted of more male participants, in particular more male caregivers, than we would have anticipated, and this may have inadvertently skewed our results toward more constrained CG relationships with patients. The literature shows that having a female CG is associated with more positive relations between the two. Gendered theories on caregiving such as the “role identity theory” also posit that embracing the role is associated with less CG burden [46]. This may explain our CG complaints about the lack of appreciation for their sacrifices, as our disproportionately male participants may not easily adjust to caregiving. Our participants did however report controlling their own emotions, something also beneficial for the patients for whom they care [32]. Furthermore, those CGs who are unwilling to have open discussions about the disease are more likely to provide low-quality care but would be less likely to join our study, having less feedback to offer and hence less resonance for the study objective [47]. The utility of providing CGs with a training course must account for their emotional state. Studies demonstrate that emotional burdens can have effects on cognition and memory, making learning more difficult. Thus, education programs that are geared toward CGs should take this into account, considering that managing emotions is critical to learning, and provide environments conducive to emotionally strained learners [48,49]. Courses for CGs should limit the cognitive load on participants through simple architecture and an easy-to-use interface for virtual modalities.

Consistent with the literature, the majority of our CG participants managed their duties while working full-time [50]. In the course of interviews, however, some noted that they took extended absences from work during the most difficult treatment stages for the patient. Those who did not take time off, however, pointed to the high levels of stress produced by managing both, considering CG duties can require a time commitment equivalent to a full-time job [50]. There exist few options for those juggling work and caregiving as unpaid leave may bring with it acute financial stress, which in turn has been associated with greater unmet needs among CGs [42]. All of our participants, however, were caring for patients with solid tumors, rather than hematologic malignancies, and the majority were older. These two characteristics are associated with less caregiver burden [51].

Our findings are limited by our sample, which was comprised of a population with strong educational attainment and relatively high incomes, as others have found that higher incomes were related to more confidence seeking health information among CGs [52]. Our participants were all English proficient, mostly white, and, with one exception, none of the CGs were caring for a patient who lacked English language skills. A few participants were immigrants, and the literature shows that immigrant caregivers have less confidence seeking health information, as do those who are non-white and have lower education levels [52]. Studies have also identified older age as being associated with a reduced likelihood of depression among CGs, whereas most of our participants were of middle-age or older [53].

While our sampling strategy aimed to recruit a diverse population, it seems that patients and CGs who had already passed the most difficult stages of treatment were more likely to participate. The distress and strain placed on social relationships, however, may be most pronounced at this stage as patients and caregivers cope with the difficult transition [32]. These participants would also be less likely to express unmet needs as these tend to decrease over time [42]. A degree of selection bias may have occurred among the healthcare providers who acted as recruiters by approaching patients and CGs who may have appeared to offer the most potentially engaging interviews. These participants may also be those that were the least burdened due to socioeconomic status and English language proficiency.

The study coincided with the COVID-19 pandemic, when infection-control measures were put in place in most healthcare settings limiting the number of entrants into hospitals. Most CGs expressed their view that this severely limited the extent to which they could provide support to their loved ones, although others appreciated the flexibility provided by virtual care. The importance of CGs to patients has led to some calls for rethinking restrictions placed on who may accompany patients during the pandemic [35]. Other participants pointed to social distancing measures as producing greater strain than would otherwise have been the case with the ability to garner more support from others. Assistance with a patient’s day-to-day activities has been shown to reduce CG burden [51]. The experience of the pandemic may have influenced the response to CG education. The isolation brought on by social distancing could reinforce the desire for in-person instruction. Participants, however, may also be attracted to the collaborative learning process associated with in-person instruction which can achieve better results [36]. Widespread experience with virtual communication platforms during the pandemic may also have increased the familiarity necessary for distance education [54].

This study indicates that with continuing unmet needs, cancer CGs would welcome greater instruction to prepare them for their role in the form of a comprehensive training course. According to participants, a program such as this would most benefit new CGs. New CGs are a challenging population to reach as they feel both unprepared and in need of information while also feeling overwhelmed. Being overwhelmed can limit the desire to learn and CG ability to partake in a course. Some participants shared that it might be helpful to “require” caregivers to complete the course to help them prioritize it and make the time to do so. With healthcare providers exercising significant influence over caregiver decision-making, they could be incorporated into the enrollment of CGs into a training course.

While the nature and modality of the course may need to be flexible to accommodate different learning preferences, goals, circumstances, and objectives, the course should be designed to alleviate the information burden on CGs while also providing them with the skills to cope emotionally with caring for a cancer patient. The lack of cancer knowledge of most participants at the beginning of their journey highlights the benefits of an educational intervention at this stage.

### Strengths and Limitations

This study is limited by its relatively small sample size. The COVID-19 pandemic made recruitment difficult, with many patients conducting virtual appointments with their oncologists and with caregivers limited in the degree to which they could accompany patients due to the stringent infection-control measures put in place at the cancer centre. As mentioned above, participants were also better educated and possessed high health literacy compared to the general population. Lastly, those most burdened caregivers were not represented due to the difficulties in securing their participation. The strength of this study is that it provides a useful snapshot of caregiver needs broadly and places them in the context of their experiences. It also addresses the avenues in which these needs can be met, based on the input of patients and CGs themselves.

## Figures and Tables

**Table 1:** Participant demographic characteristics. Health literacy score; computer literacy score.

Variable	Patient	Caregiver
Age (mean; range)	53.8; 43–70	53.1; 29–71
Gender	4–Males 2–Females	5–Males 2–Females
Race/Ethnicity	5–White/Caucasian/European 1–Other (“biracial–black Caribbean and white Canadian”	5–White/Caucasian/European 1–Arab/west-Asian 1–Other (“half white/Asian”)
Highest level of schooling completed	4–college/university 1–high school 1–post-graduate school	5–college/university 1–post-graduate school
Household income in the last year *	3–More than CAD 100,000 1–CAD 80,000–99,999 1– < CAD 400,000 1–Prefer not to say	3–More than CAD 100,000 1 CAD 80,000–99,999 2–CAD 60,000–79,999 1–CAD 40,000–59,999
Marital status	2–Single, never married 4–Married, common law	2–Single, never married 3–Married, common law
Main work-related activity	3–Getting disability payment 2–Working (part-time or full-time) 1–Other (retired)	5–Working (part-time or full-time) 1–Unemployed 1–Other (retired)
Living arrangements	1–Alone 4–With partner 2–With children	1–Alone1–With roommates 2–With parents 2–With partner 1–With children
Cancer type	5–Head and Neck (nose, mouth, throat) 1–Other (breast and thyroid)	6–Head and Neck (nose, mouth, throat) 1–Other (breast)
Treatments received	2–Surgery 5–Radiation 4–Chemotherapy	2–Surgery 6–Radiation 4–Chemotherapy
Health literacy score (mean)	45.8/64	52.7/64
Computer literacy score (mean)	42.7/48	46.7/48

* All figures in CAD.

## Data Availability

The data presented in this study are available on request from the corresponding author.

## Supplementary Materials

The following supporting information can be downloaded at: https://www.mdpi.com/article/10.3390/curroncol30040291/s1, Supplementary File S1 (draft outline).

## References

[1] Cancer Cases Expected to Soar 40 per Cent by 2030. CTV News (2015). https://www.ctvnews.ca/health/cancer-cases-expected-to-soar-40-per-cent-by-2030-1.2392833.

[2] Cancer: Key Statistics. https://www.who.int/cancer/resources/keyfacts/en/.

[3] Family Caregiving: What Are the Consequences?. https://www150.statcan.gc.ca/n1/pub/75-006-x/2013001/article/11858-eng.htm.

[4] Hollander M.J., Liu G., Chappell G.L.A.N.L. (2009). Who Cares and How Much? The Imputed Economic Contribution to the Canadian Healthcare System of Middle-Aged and Older Unpaid Caregivers Providing Care to The Elderly. World Health Popul..

[5] Bastawrous M. (2013). Caregiver burden—A critical discussion. Int. J. Nurs. Stud..

[6] Van Ryn M., Sanders S., Kahn K., Van Houtven C., Griffin J.M., Martin M., Atienza A.A., Phelan S., Finstad D., Rowland J. (2010). Objective burden, resources, and other stressors among informal cancer caregivers: A hidden quality issue?. Psycho-Oncology.

[7] McCorkle R., Ercolano E., Lazenby M., Schulman-Green D., Schilling L.S., Lorig K., Wagner E.H. (2011). Self-management: Enabling and empowering patients living with cancer as a chronic illness. CA A Cancer J. Clin..

[8] Giuliani M., Milne R., Puts M., Sampson L., Kwan J., Le L., Alibhai S., Howell D., Abdelmutti N., Liu G. (2016). The Prevalence and Nature of Supportive Care Needs in Lung Cancer Patients. Curr. Oncol..

[9] Maqbool T., Agarwal A., Sium A., Trang A., Chung C., Papadakos J. (2016). Informational and Supportive Care Needs of Brain Metastases Patients and Caregivers: A Systematic Review. J. Cancer Educ..

[10] Papadakos J., Bussière-Côté S., Abdelmutti N., Catton P., Friedman A.J., Massey C., Urowitz S., Ferguson S.E. (2012). Informational needs of gynecologic cancer survivors. Gynecol. Oncol..

[11] Papadakos J., McQuestion M., Gokhale A., Damji A., Trang A., Abdelmutti N., Ringash J. (2017). Informational Needs of Head and Neck Cancer Patients. J. Cancer Educ..

[12] Papadakos J., Urowitz S., Olmstead C., Friedman A.J., Zhu J., Catton P. (2014). Informational needs of gastrointestinal oncology patients. Heal. Expect.

[13] Moghaddam N., Coxon H., Nabarro S., Hardy B., Cox K. (2016). Unmet care needs in people living with advanced cancer: A systematic review. Support. Care Cancer.

[14] Girgis A., Lambert S., Johnson C., Waller A., Currow D. (2013). Physical, Psychosocial, Relationship, and Economic Burden of Caring for People With Cancer: A Review. J. Oncol. Pr..

[15] Navaie-Waliser M., Feldman P.H., Gould D.A., Levine C., Kuerbis A.N., Donelan K. (2002). When the Caregiver Needs Care: The Plight of Vulnerable Caregivers. Am. J. Public Health.

[16] Wang T., Molassiotis A., Chung B.P.M., Tan J.-Y. (2018). Unmet care needs of advanced cancer patients and their informal caregivers: A systematic review. BMC Palliat. Care.

[17] Paek M.-S., Nightingale C., Tooze J., Milliron B.-J., Weaver K., Sterba K. (2018). Contextual and stress process factors associated with head and neck cancer caregivers’ physical and psychological well-being. Eur. J. Cancer Care.

[18] Litzelman K., Kent E.E., Mollica M., Rowland J.H. (2016). How Does Caregiver Well-Being Relate to Perceived Quality of Care in Patients With Cancer? Exploring Associations and Pathways. J. Clin. Oncol..

[19] Wittenberg E., Borneman T., Koczywas M., Del Ferraro C., Ferrell B. (2017). Cancer Communication and Family Caregiver Quality of Life. Behav. Sci..

[20] Papadakos J., Samoil D., Umakanthan B., Charow R., Jones J.M., Matthew A., Nissim R., Sayal A., Giuliani M.E. (2021). What are we doing to support informal caregivers? A scoping review of caregiver education programs in cancer care. Patient Educ. Couns..

[21] Blusi M., Kristiansen L., Jong M. (2014). Exploring the influence of Internet-based caregiver support on experiences of isolation for older spouse caregivers in rural areas: A qualitative interview study. Int. J. Older People Nurs..

[22] Berry L.L., Dalwadi S.M., Jacobson J.O. (2017). Supporting the Supporters: What Family Caregivers Need to Care for a Loved One With Cancer. J. Oncol. Pr..

[23] Fundytus A., Hopman W., Hammad N., Biagi J., Sullivan R., Vanderpuye V., Seruga B., Lopes G., Sengar M., Brundage M. (2018). Medical Oncology Workload in Canada: Infrastructure, Supports, and Delivery of Clinical Care. Curr. Oncol..

[24] Kent E.E., Rowland J.H., Northouse L., Litzelman K., Chou W.-Y.S., Shelburne N., Timura C., O’Mara A., Huss K. (2016). Caring for caregivers and patients: Research and clinical priorities for informal cancer caregiving. Cancer.

[25] Creswell J.W. (2013). Qualitative Inquiry and Research Design: Choosing among Five Approaches.

[26] Hsiung P.-C. (2008). Teaching Reflexivity in Qualitative Interviewing. Teach. Sociol..

[27] Lorini C., Lastrucci V., Mantwill S., Vettori V., Bonaccorsi G. (2019). Measuring health literacy in Italy: A validation study of the HLS-EU-Q16 and of the HLS-EU-Q6 in Italian language, conducted in Florence and its surroundings. Ann. Dell’istituto Super. Sanità.

[28] Boot W.R., Charness N., Czaja S.J., Sharit J., Rogers W.A., Fisk A.D., Mitzner T.L., Lee C.C., Nair S. (2013). Computer Proficiency Questionnaire: Assessing Low and High Computer Proficient Seniors. Gerontologist.

[29] Kuzel A.J., Crabtree B.F., Miller W.L. (1992). Sampling in Qualitative Inquiry. Doing Qualitative Research.

[30] Bazely P., Jackson K., Seaman J. (2010). Nvivo Qualitative Data Analysis Software.

[31] Lee J.E., Shin D.W., Cho J., Yang H.K., Kim S.Y., Yoo H.S., Jho H.J., Shin J.Y., Cho B., Park K. (2015). Caregiver burden, patients’ self-perceived burden, and preference for palliative care among cancer patients and caregivers. Psycho-Oncology.

[32] Chen S.-C., Chiou S.-C., Yu C.-J., Lee Y.-H., Liao W.-Y., Hsieh P.-Y., Jhang S.-Y., Lai Y.-H. (2016). The unmet supportive care needs—What advanced lung cancer patients’ caregivers need and related factors. Support. Care Cancer.

[33] Kim Y., Given B.A. (2008). Quality of life of family caregivers of cancer survivors. Cancer.

[34] Kuo H.J., Chun J., Lee G., Curtiss S. (2021). Competencies and preferences of online psycho-education for caregivers of transition-aged autistic youth. J. Enabling Technol..

[35] Alewine C., Ahmad M., Shea R. (2021). Caregiver Exclusion in the Age of COVID: Fighting Cancer With Half the Team. J. Clin. Oncol..

[36] Conklin J., Lusk E., Harris M., Stolee P. (2013). Knowledge brokers in a knowledge network: The case of Seniors Health Research Transfer Network knowledge brokers. Implement. Sci..

[37] Robeson P., Dobbins M., DeCorby K. (2008). Life as a knowledge broker in public health. J. Can. Health Libr. Assoc..

[38] Lambert S.D., Loiselle C.G. (2007). Health Information—Seeking Behavior. Qual. Health Res..

[39] Monteiro F. (2013). Family Reliance on Physicians’ Decisions in Life-Sustaining Treatments in Acute-on-Chronic Respiratory Diseases in a Respiratory ICU: A Single-Center Study. Respir. Care.

[40] Lund L., Ross L., Petersen M.A., Grønvold M. (2014). The interaction between informal cancer caregivers and health care professionals: A survey of caregivers’ experiences of problems and unmet needs. Support. Care Cancer.

[41] Moore C., Hassett D., Dunne S. (2021). Health literacy in cancer caregivers: A systematic review. J. Cancer Surviv..

[42] Girgis A., Lambert S.D., McElduff P., Bonevski B., Lecathelinais C., Boyes A., Stacey F. (2012). Some things change, some things stay the same: A longitudinal analysis of cancer caregivers’ unmet supportive care needs. Psycho-Oncology.

[43] Kim Y., Wellisch D.K., Spillers R.L., Crammer C. (2007). Psychological distress of female cancer caregivers: Effects of type of cancer and caregivers’ spirituality. Support. Care Cancer.

[44] Rosland A.-M., Heisler M., Choi H., Silveira M.J., Piette J.D. (2010). Family influences on self-management among functionally independent adults with diabetes or heart failure: Do family members hinder as much as they help?. Chronic Illn..

[45] Yuen E., Knight T., Dodson S., Chirgwin J., Busija L., Ricciardelli L.A., Burney S., Parente P., Livingston P.M. (2017). Measuring cancer caregiver health literacy: Validation of the Health Literacy of Caregivers Scale-Cancer (HLCS-C) in an Australian population. Health Soc. Care Community.

[46] Kim Y., Mitchell H., Ting A. (2018). Application of psychological theories on the role of gender in caregiving to psycho-oncology research. Psycho-Oncology.

[47] Wittenberg E., Goldsmith J.V., Kerr A.M. (2019). Variation in health literacy among family caregiver communication types. Psycho-Oncology.

[48] Madsgaard A., Smith-Strøm H., Hunskår I., Røykenes K. (2021). A rollercoaster of emotions: An integrative review of emotions and its impact on health professional students’ learning in simulation-based education. Nurs. Open.

[49] Lei X. (2022). The impact of emotion management ability on learning engagement of college students during COVID-19. Front. Psychol..

[50] Deshields T.L., Applebaum A.J. (2015). The time is now: Assessing and addressing the needs of cancer caregivers. Cancer.

[51] Ge L., Mordiffi S.Z. (2017). Factors Associated With Higher Caregiver Burden Among Family Caregivers of Elderly Cancer Patients. Cancer Nurs..

[52] Bangerter L.R., Griffin J., Harden K., Rutten L.J. (2019). Health Information–Seeking Behaviors of Family Caregivers: Analysis of the Health Information National Trends Survey. JMIR Aging.

[53] Geng H.-M., Chuang D.-M., Yang F., Yang Y., Liu W.-M., Liu L.-H., Tian H.-M. (2018). Prevalence and determinants of depression in caregivers of cancer patients. Medicine.

[54] Silva O., Sousa A., Nunes J. (2021). Technology’s Impacts in the Students of Higher Education in the Covid-19 Pandemic Period. Perspectives and Trends in Education and Technology: Selected Papers from ICITED 2021.

