# Supplementary material for: Assessing the Comprehensive Training Needs of Informal Caregivers of Cancer Patients: A Qualitative Study
Source: Curr Oncol. 2023 Mar 29;30(4):3845–58. doi: 10.3390/curroncol30040291 (PMC10137188; doi:10.3390/curroncol30040291)
Supplement: Supplementary file 1 [file curroncol-30-00291-s001.zip › curroncol-2257195-supplementary.pdf]

Supplementary File S1. Draft Caregiver Curriculum Outline

Figure S1. Managing the medical aspects of illness.

How to Manage...	How to Maintain a Healthy Lifestyle	How to Perform Clinical Tasks
• Pain<br>• Fatigue<br>• Weakness<br>• Hair loss<br>• Nausea and vomiting<br>• Changes in appetite and nutrition requirements<br>• Maintaining a normal calorie intake<br>• Managing anorexia/weight-changes<br>• Changes in bowel habits<br>• Diarrhea<br>• Constipation<br>• Incontinence/frequent urination<br>• Changes in behaviour, character, or cognition• Changes in sleep quality and quantity<br>• The side effects of medication<br>• The long-term consequences of treatment<br>• The impact of cancer on fertility<br>• How to manage changes in sexual intimacy<br>• How to engage in safe sexual	• How to exercise and engage in physical activity during treatment<br>• Managing the risk of falling<br>• Maintaining optimal mobility<br>• The benefits of quitting smoking	• Post-surgery care<br>• Infection prevention and control<br>• Hand hygiene<br>• How to lower your risk of infection<br>• Wound care<br>• Lacerations & abrasions<br>• Cleaning your wound<br>• Packing your wound<br>• Dressing and bandaging<br>• Caring for your surgical drain<br>• Caring for a central line<br>• Caring for a feeding tubes<br>• Care for an ostomy<br>• Care for a tracheostomy

practices (e.g. when to use condoms, can you get partner sick)<br>• Exploring alternative fertilization options (e.g. freezing your eggs)<br>• Coping with impotence • Cancer and menopause		

Figure S2. Managing changes in roles and relationships to accommodate illness.

How to Manage Changes in your Relationship with...	Developing Adaptive Communication Strategies	Obtaining Psychosocial Support
• Your spouse/significant other<br>• Individuals in the workplace<br>• Your friends and family (e.g. children)<br>• Yourself (e.g. how to define your identity in the face of a role transition)	• How to make decisions as a team/patient caregiver pair<br>• How to share your thoughts and feelings<br>• How to demonstrate active listening (e.g. validating feelings, demonstrating understanding)<br>• How to minimize un-adaptive coping (e.g. nagging, criticizing)<br>• How to communicate with medical staff<br>• How to talk to your children about your cancer	• How to find and ask for help from others<br>• How to access counselling services at PMCC<br>• How to connect with others who share a similar experience with others (e.g. support group services at PMCC)<br>• How to connect with the patient beyond providing care (e.g. activities offered at PMCC, like art and exercise classes, that family members can do together)

	• How to talk to your children about death and dying	

Figure S3. Managing the psychological consequences of illness through use of problem-solving coping strategies.

How to Cope with...	Mood and Affect Management Techniques	Developing Positive Self-Schema
• Stress<br>• Anxiety<br>• Depression<br>• Grief/loss<br>• Hopelessness<br>• Worry/fear (e.g. of cancer re-occurrence, of treatment failure)<br>• Anger/guilt<br>• Changes in the patient's body image<br>• Changes in the patient's self image<br>• Changes in the patient's thinking and cognition (e.g. delirium)<br>• Unpleasant emotions you have due to the patient's cancer diagnosis<br>• The emotional reactions of others (e.g.	• Maintaining optimism<br>• Mindfulness and relaxation<br>• Addressing negative beliefs about cancer	• Strategies to improve self-esteem and self- efficacy<br>• Coping with loss of personal control<br>• Developing self-compassion

friends/family) to the patient’s cancer diagnosis<br>• Uncertainty about the future
